# *robustica*: customizable robust independent component analysis

**DOI:** 10.1186/s12859-022-05043-9

**Published:** 2022-12-05

**Authors:** Miquel Anglada-Girotto, Samuel Miravet-Verde, Luis Serrano, Sarah A. Head

**Affiliations:** 1 Centre for Genomic Regulation (CRG), The Barcelona Institute of Science and Technology, Barcelona, Spain; 2 Universitat Pompeu Fabra (UPF), Barcelona, Spain; 3 ICREA, Pg. LLuís Companys 23, 08010 Barcelona, Spain

**Keywords:** Bioinformatics, Independent component analysis, Clustering, Unsupervised learning, Low-grade glioma, Python

## Abstract

**Background:**

Independent Component Analysis (ICA) allows the dissection of omic datasets into modules that help to interpret global molecular signatures. The inherent randomness of this algorithm can be overcome by clustering many iterations of ICA together to obtain robust components. Existing algorithms for robust ICA are dependent on the choice of clustering method and on computing a potentially biased and large Pearson distance matrix.

**Results:**

We present *robustica*, a Python-based package to compute robust independent components with a fully customizable clustering algorithm and distance metric. Here, we exploited its customizability to revisit and optimize robust ICA systematically. Of the 6 popular clustering algorithms considered, *DBSCAN* performed the best at clustering independent components across ICA iterations. To enable using Euclidean distances, we created a subroutine that infers and corrects the components’ signs across ICA iterations. Our subroutine increased the resolution, robustness, and computational efficiency of the algorithm. Finally, we show the applicability of *robustica* by dissecting over 500 tumor samples from low-grade glioma (LGG) patients, where we define two new gene expression modules with key modulators of tumor progression upon *IDH1* and *TP53* mutagenesis.

**Conclusion:**

*robustica* brings precise, efficient, and customizable robust ICA into the Python toolbox. Through its customizability, we explored how different clustering algorithms and distance metrics can further optimize robust ICA. Then, we showcased how *robustica* can be used to discover gene modules associated with combinations of features of biological interest. Taken together, given the broad applicability of ICA for omic data analysis, we envision *robustica* will facilitate the seamless computation and integration of robust independent components in large pipelines.

**Supplementary Information:**

The online version contains supplementary material available at 10.1186/s12859-022-05043-9.

## Background

Independent Component Analysis (ICA) is a matrix factorization method that dissects a mixture of signals into a predefined number of additive independent sources or components. ICA finds sets of statistically independent components by minimizing their mutual information [1]. In biology, ICA has a wide range of applications such as defining functional modules, removing technical noise, feature engineering, unsupervised cell type deconvolution, single-cell trajectory inference, or multi-omic analysis (reviewed in [2]). Thanks to its information-theoretic objective function, ICA results in components that provide a simpler, more reproducible, and more biologically relevant interpretation than other popular matrix factorization methods such as Principal Component Analysis (PCA) or Non-negative Matrix Factorization (NMF) [2–7].

*FastICA* [8], one of the most widespread algorithms used to perform ICA, starts with a random initialization to decompose the data matrix into a source matrix and a mixing matrix of non-Gaussian independent components (Additional file 1: Fig. S1A). In 2003, Hymberg and Hyvärinen [9] developed *Icasso* to address the inherent randomness of *FastICA,* by running *FastICA* multiple times and clustering the components of source matrices across all runs (Additional file 1: Fig. S1B). This clustering step involves two key choices affecting its computational efficiency and the final robust components: the distance metric and the clustering algorithm. Current implementations require pre-computing a potentially large Pearson distance square matrix to cluster components across ICA runs regardless of their different sign and order resulting from *FastICA*’s randomness [9–12]. However, correlation-based metrics are sensitive to outliers and non-Gaussian distributions as independent components, which may lead to calculating imprecise weights [13]. Additionally, more recently developed clustering algorithms could potentially improve the efficiency and quality of robust ICA towards distilling technically unbiased gene modules.

Here, we developed *robustica*, the first Python package to carry out robust ICA with a fully customizable clustering metric and algorithm based on the powerful library *scikit-learn* [14]. By leveraging the customizability of our package to revisit and optimize the clustering step of the *Icasso* algorithm, we improved its resolution, robustness, and computational efficiency. Finally, as a case study, we dissected gene expression signatures from patients with low-grade glioma (LGG) and found two sets of genes related to the mechanisms by which mutations in *IDH1* and *TP53* lead to tumor progression.

## Implementation

*robustica* is written in Python under the open-source 3-Clause BSD license. The source code and documentation are freely available at https://github.com/CRG-CNAG/robustica. Additionally, all scripts to reproduce the work presented are available at https://github.com/MiqG/publication_robustica.

The algorithm to carry out robust ICA is controlled by the main class *RobustICA*. After instantiation, one can use the *fit* method on a data matrix to run the *FastICA* algorithm multiple times and cluster the resulting independent components with the desired number of components and clustering distance metric and algorithm. Subsequently, one can recover the computed source and mixing matrices through the *transform* method.

To facilitate the customizability and seamless integration of the algorithm, the user can specify all the parameters related to every step of the robust ICA algorithm as similarly as possible to the core *FastICA* class already available in *scikit-learn*. For this reason, we only required 6 more arguments labeled using the “robust_” prefix. These arguments determine: the number of times to run *FastICA* before clustering (*robust_runs*), whether to use our subroutine to infer and correct the signs of the components across *FastICA* runs (*robust_infer_signs*), the custom clustering algorithm class (*robust_method*), the keywords to pass to the clustering algorithm class (*robust_kws*), whether to speed up the clustering step using PCA to reduce the dimensions of the independent components across all runs (*robust_dimreduce*) and, when the distance needs to be precomputed, the function to use to compute pairwise distances in the clustering step (*robust_precompdist_func*).

## Results

### *robustica* enables systematic evaluation of clustering algorithms to perform robust ICA

With *robustica,* one can fully customize the clustering method to use as long as they follow *scikit-learn* conventions [14]. We purposefully included this feature to compare how 6 different popular clustering algorithms perform at finding robust components (see Methods). As a benchmark, we dissected the gene expression signatures from 43 transcriptomic datasets containing different numbers of samples and features (Additional file 1: Fig. S2; Additional file 1: Table S1) into 100 components with 100 ICA runs and selected different clustering algorithms to compute their robust components. We then evaluated the performance of the different clustering algorithms by measuring their run time, memory usage, and silhouette scores to quantify how similar each component is to the components in the cluster compared to the components assigned to other clusters. Exceptionally, we could not measure the silhouette scores for the density-based algorithm *AffinityPropagation* as it did not converge for any of the datasets using its default parameters.

**Fig. 1:**
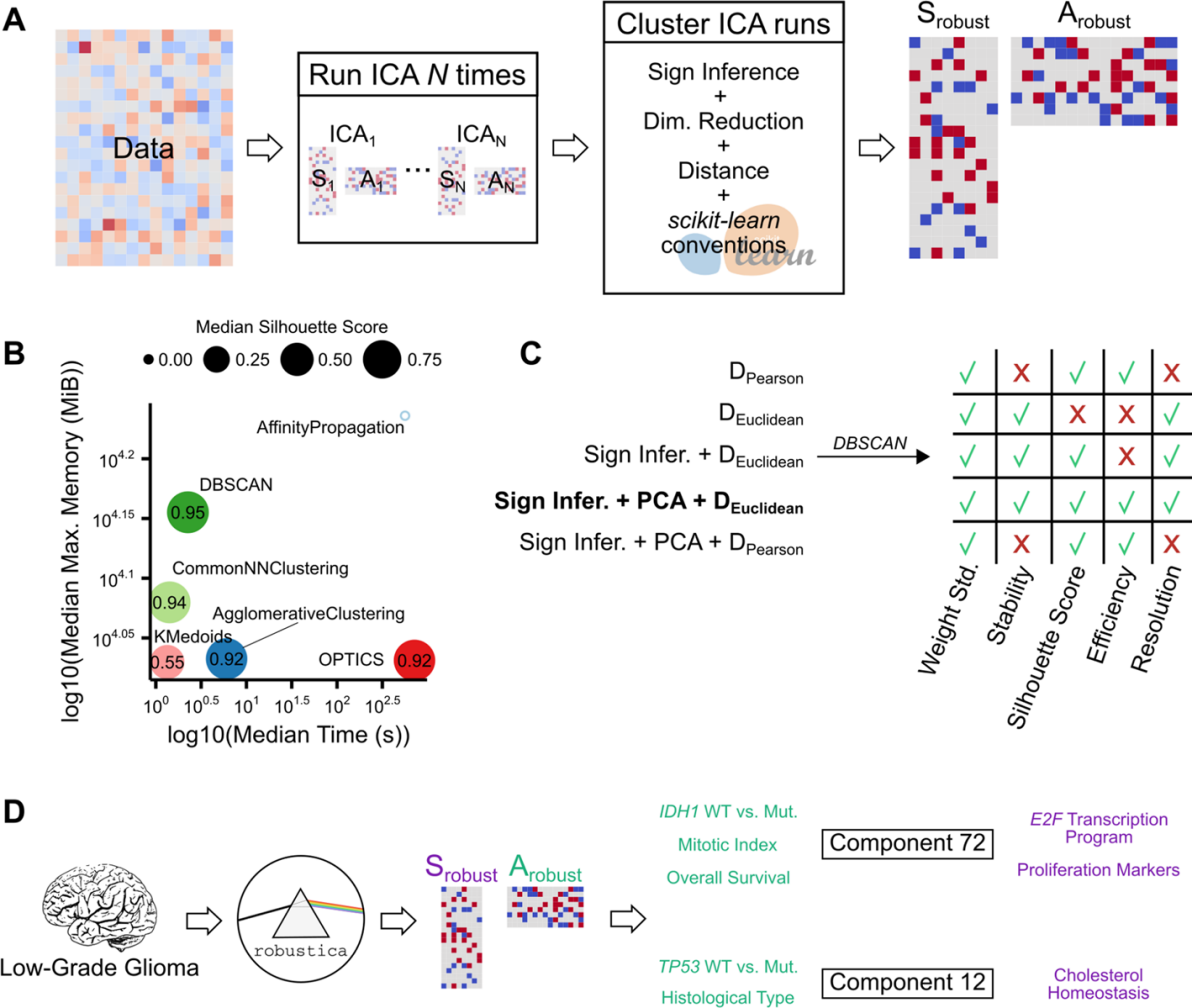
Development and implementation of *robustica* to carry out robust Independent Component Analysis (ICA). **A** *robustica* enables fully customized robust ICA. We built *robustica* following *scikit-learn*’s programming conventions to enable full control of the iterative and clustering steps, facilitating customization and optimization. In particular, *robustica* includes (i) a subroutine to infer and correct the signs of components across ICA runs that improves the precision and efficiency of the clustering step by adapting the use of Euclidean distance metrics and (ii) the option to speed up the computation of pairwise distances by compressing the feature space with PCA. **B** Comparison of clustering algorithms for 43 different transcriptomic datasets. Median time (x-axis) and the median maximum memory usage (y-axis) for each clustering algorithm to cluster 100 ICA runs with 100 components each. Dot sizes indicate median silhouette scores (the larger the better, with a maximum of 1). Note that the *AffinityPropagation* algorithm is represented as an empty dot due to silhouette scores being non-computable as convergence was not reached with the tested parameters. **C** Development steps to improve the resolution and efficiency of robust ICA through a sign inference-and-correction subroutine combined with PCA and Euclidean distances, using the 43 datasets. **D** Case study workflow for *robustica* dissecting > 500 LGG patients’ tumor samples into 100 robust independent components. Components 72 and 12 were simultaneously associated with multiple sample features (green) and contained genes known to be mechanistically associated with known mechanisms of tumor progression. *scikit-learn* logo adapted from https://commons.wikimedia.org/wiki/File:Scikit_learn_logo_small.svg (3-clause BSD license)

We considered algorithms with either a predefined *k* (k-based) or a free (density-based) number of clusters. Overall, k-based clustering algorithms -*AgglomerativeClustering, KMedoids*- were fast and memory-efficient but resulted in lower silhouette scores than density-based algorithms -*CommonNNClustering, DBSCAN, OPTICS*- (Fig. 1B; Additional file 1: Fig. S3; Additional file 1: Tables S2–S4). K-based algorithms assign a cluster to all observations producing lower silhouette scores compared to density-based algorithms that discriminate as “noise” ambiguous observations. (Additional file 1: Fig. S4A, B). Consequently, density-based algorithms only consider highly similar independent components across ICA runs to compute robust components while omitting divergent independent components; however, they return an a priori unpredictable number of robust independent components depending on the *min_samples* parameter (Additional file 1: Fig. S4C). This feature helps to interpret the biological relevance of robust independent components that are not derived from the inherent randomness of the *FastICA* algorithm. Finally, we studied how well clustering algorithms scale with each dataset's different numbers of samples and genes (as features). As expected, the number of initial samples used to run ICA multiple times is not associated with any performance metric, and memory usage and silhouette scores increased as we dissected datasets with more features (Additional file 1: Fig. S5A). On the other hand, increasing the number of genes increased memory usage and silhouette scores keeping the clustering time the same (Additional file 1: Fig. S5B). Finally, as a sanity check, we confirmed that our conclusions remained the same regardless of using Pearson-based silhouette scores instead of the default Euclidean-based silhouette scores implemented above (Additional file 1: Fig. S6A–C). In fact, default Euclidean-based silhouette scores offer better discrimination of high-scoring clusters than Pearson-based silhouette scores which rapidly saturate when clusters reach high values (Additional file 1: Fig. S6D).

As an example, we studied in detail the results from the robust ICA dissecting > 250 *E. coli* gene expression signatures from Sastry et al. [15]. On the computational efficiency side, distance and centroid computations are the most time- and memory-intensive steps, respectively (Additional file 1: Fig. S7). By comparing the two first principal components computed from all 10,000 ICA runs we conclude observations belonging to highly crowded regions are clustered together in density-based algorithms leaving out unstable independent components. In fact, DBSCAN clustered the highest number of points within low-quality clusters, which may lead to precise robust independent components with at least 50 components in this benchmark (Additional file 1: Fig. S8; Additional file 1: Table S5). Finally, we confirmed that *robustica* reproduces Sastry et al. [15]’s robust independent components when using the same parameters for the clustering step with *DBSCAN* (Additional file 1: Fig. S9; Additional file 1: Tables S6 and S7).

All in all, density-based clustering algorithms help to distill high-quality independent components aiding the characterization and interpretation. In particular, the *DBSCAN* algorithm showed the best performance tradeoff taking a median of 2.26 s and 14,295 MiB of maximum memory usage to obtain clusters with the highest silhouette scores across the 43 datasets considered (*median* = *0.95*) (Fig. 1B). Therefore, we selected *DBSCAN* for subsequent steps as it offers the best tradeoff between computational performance, quality of the robust components, and flexible selection of robust components across separate ICA runs.

### Clustering ICA runs with Euclidean distances improves the resolution and reproducibility of robust ICA

After running ICA multiple times, the *Icasso* algorithm computes a pairwise Pearson distance matrix among all independent components across all ICA runs (Additional file 1: Fig. S1B). Using Pearson distance helps cluster together the most similar components regardless of the sign and order that results from running *FastICA* multiple times. However, the sensitivity of Pearson distance to the non-Gaussian distributions of independent components may bias weights in robust independent components. Hence, clustering metrics such as Euclidean distance may help bypass this problem. To implement Euclidean distance, first, we need to make sure that similar independent components have the same sign before clustering them. We tackled this with a simple subroutine to infer and correct the sign of the components across ICA runs to enable using Euclidean distances (see Methods). In addition, we compressed the feature space of all ICA runs through PCA to reduce the overall run time and memory usage while maintaining the same performance (Additional file 1: Fig. S10A; Additional file 1: Tables S8 and S9).

Computing robust independent components with Pearson distances was time and memory efficient and produced fewer components of high Euclidean- or Pearson-based silhouette scores and weights of low standard deviation than Euclidean distances (Additional file 1: Fig. S10A, Additional file 1: Fig. S11). Interestingly, our subroutine based on sign inference-and-correction and Euclidean distances leads to a higher number of robust independent components with a mild increase in the standard deviation of their weights. While these differences may make Euclidean distances less robust to random noise than Pearson distances (Additional file 1: Fig. S10B; Additional file 1: Table S10), Euclidean distances lead to stable gene modules when using fewer ICA runs to recover most of the gene modules defined using 100 ICA runs to compute robust components (Additional file 1: Fig. S10C; Additional file 1: Table S11). Surprisingly, with Pearson distances, *DBSCAN* tends to output a lower number of robust independent components as we increase the number of ICA runs considered while remaining constant for Euclidean distances. Euclidean-based distances assure a high reproducibility of gene modules regardless of the number of ICA runs used (Additional file 1: Fig. S10C).

Altogether, our sign inference-and-correction subroutine creates high-quality and stable robust independent components by enabling us to efficiently and reproducibly cluster independent components across ICA runs using Euclidean distances (Fig. 1C).

### *robustica* recovers a gene expression module with the key regulators of tumor progression in LGG mechanistically associated with mutations in *IDH1* and *TP53*

As a case study, we dissected gene expression profiles from 530 LGG tumor samples from The Cancer Genome Atlas (TCGA) using different clustering metrics. Our sign inference-and-correction subroutine combined with Euclidean distances produced a total of 94 robust independent components, 31 more than Pearson distances with a similar overall quality and computational efficiency (Additional file 1: Fig. S12; Additional file 1: Fig. S13A–C; Additional file 1: Tables S12–S18). Combined with the previous results, this may indicate that Euclidean distances confer a higher resolution than Pearson distances for the *DBSCAN* clustering algorithm as extra robust independent components tend to have weights with higher standard deviation and lower silhouette scores than components mapped in both approaches (Additional file 1: Fig. S13D). We then wondered whether components uniquely identified through our approach are also likely to carry biologically-relevant information.

Missense mutations in the isocitrate dehydrogenase (*IDH1*) and tumor protein P53 (*TP53*) are highly common among the 530 LGG tumor samples analyzed (Additional file 1: Fig. S14; Additional file 1: Table S19). LGGs are characterized by mutations in the *IDH1* enzyme that decrease tumor aggressiveness by indirectly inhibiting the *E2F* transcription program, an important switch controlling homeostasis and tumorigenesis [16–18]. On the other hand, upon mutation, *TP53* loses its tumor-suppressor function and induces tumorigenesis through genomic instability [19]*. TP53* has a central role in cancer and is involved in many processes, among them, cholesterol homeostasis through the Hippo and mevalonate pathways are involved in cancerous transformation [20, 21]. Therefore, we explored whether gene modules could recover known molecular mechanisms associated with *IDH1* or *TP53* mutations. We focused on two robust independent components (i.e. 72 and 12), identified either through both clustering metrics or only through Euclidean distances, respectively. From the properties considered, weights in component 72 were highly associated with *IDH1* mutation status, expression-based indices of cell proliferation, and patient overall survival probability (Additional file 1: Fig. S15A–D; Additional file 1: Tables S20 and S21). Conversely, weights in component 12 were highly associated with *TP53* mutation status and tumor histological type (Additional file 1: Fig. S5A–D; Additional file 1: Tables S20 and S21).

Finally, we related these sample-level traits to the gene expression signatures by defining two gene modules of 420 (module 72) and 159 (module 12) genes using the extreme weights of the corresponding component (Additional file 1: Fig. S15E; Additional file 1: Table S22) (see Methods). Module 72 was enriched in proliferation-related biological processes and contained 7 out of the 9 genes used to compute the mitotic index [22] and known proliferation markers as *MKI67* [23]. Interestingly, our gene module also included 4 *E2F* transcription factors (*E2F1, E2F2, E2F7, E2F8*) and was enriched with 98 targets of *E2F*s (Additional file 1: Fig. S15F; Additional file 1: Table S23). On the other hand, module 12 was enriched in terms related to cholesterol homeostasis (Additional file 1: Fig. S15F; Additional file 1: Table S23) confirming the relationship between *TP53* mutation status and cholesterol metabolism.

With this, we demonstrate the utility of *robustica* and our revisited *Icasso* algorithm to identify gene sets whose unique transcriptional signature in LGG is associated with genotypes and phenotypes of interest, demonstrating the biological applicability of this approach (Fig. 1D).

## Conclusions

We created *robustica*, a new Python package built on top of *scikit-learn* that enables performing precise, efficient, and customizable robust ICA seamlessly. Using different clustering algorithms and distance metrics, we tested whether robust ICA could be further optimized. Our sign correction subroutine improved the resolution, robustness, and computational efficiency of the clustering step. As an example, we explored how the gene modules generated with *robustica* from transcriptomic profiles of LGG patients are associated with multiple markers of the disease’s progression simultaneously. Overall, *robustica* makes high-performance robust ICA more accessible to potentially analyze any omic data type and facilitates its incorporation in larger computational pipelines.

## Supplementary Information

**Additional file 1**. Document containing Methods, Supplementary Figures, and Supplementary Tables.

## Data Availability

Source code and documentation: https://github.com/CRG-CNAG/robustica. Analysis pipeline in this paper: https://github.com/MiqG/publication_robustica. Supplementary tables were made available through the Zenodo online repository: https://zenodo.org/record/6833937.

## Abbreviations

ICA: Independent Component Analysis
LGG: Low-Grade Glioma
PCA: Principal Component Analysis
NMF: Non-negative Matrix Factorization
TCGA: The Cancer Genome Atlas
IDH1: Isocitrate dehydrogenase
TP53: Tumor protein P53

## Availability and requirements

Project name: *robustica*

Project home page: https://github.com/CRG-CNAG/robustica

Operating system(s): Platform Independent

Programming language: Python

Other requirements: *numpy 1.19.2, pandas 1.1.2, scikit-learn 0.23.2, scikit-learn-extra 0.2.0, joblib 1.0.1, tqdm 4.59.0*

License: BSD 3-Clause

Any restrictions to use by non-academics: None
