# Supplementary material for: robustica: customizable robust independent component analysis
Source: BMC Bioinformatics. 2022 Dec 5;23:519. doi: 10.1186/s12859-022-05043-9 (PMC9721028; doi:10.1186/s12859-022-05043-9)
Supplement: Supplementary file 1 — Additional file 1. Document containing Methods, Supplementary Figures, and Supplementary Tables. [file 12859_2022_5043_MOESM1_ESM.docx]

# **METHODS**

All scripts to reproduce the work presented are available at <https://github.com/MiqG/publication_robustica>.

## **Data download and preprocessing**

We downloaded the 278 gene expression signatures for 3’923 genes in *E. coli* from Sastry (2019)[^1^](https://www.zotero.org/google-docs/?XaM0xH) ’s Supplementary Data 1. Following the author’s procedure, we normalized the expression signatures with respect to controls (“control__wt_glc__1” and “control__wt_glc__2”) by subtracting the mean gene expression of the controls from the rest of the signatures.

We used the XenaBrowser[^2^](https://www.zotero.org/google-docs/?Rh38AL) to obtain the pan-cancer log-normalized gene expression signatures, somatic mutation, and clinical information in samples from patients in The Cancer Genome Atlas (TCGA) for the cancer types listed in Supplementary Table 1.

We downloaded v8 GTEx TPM-normalized gene expression signatures and sample information from their website (<https://gtexportal.org/home/datasets>).

We computed the mitotic index for each sample by averaging the log-normalized expression levels of 9 genes (*CDKN3*, *ILF2*, *KDELR2*, *RFC4*, *TOP2A*, *MCM3*, *KPNA2*, *CKS2*, and *CDK1*) as described in Yang (2016)[^3^](https://www.zotero.org/google-docs/?5j26Bw).

## **Comparing different clustering algorithms to compute robust independent components**

We used the preprocessed datasets to compare how different clustering algorithms perform in terms of computing time, memory usage and cluster average silhouette scores, and cluster weight standard deviation. We followed the *Icasso* procedure described in Himberg and Hyvärinen (2003)[^4^](https://www.zotero.org/google-docs/?sjL3so) and modified the clustering step. First, we standardized the gene expression matrix across genes (rows). Then, we used *robustica* to run *FastICA* 100 times with 100 components and default parameters. We saved the source (S) and mixing (A) matrices generated across the runs to ensure that we used the same inputs for the clustering step. Subsequently, we created a new instance of *robustica* to cluster the components across the S matrices using the precomputed distance matrix based on absolute Pearson correlations as input:

$d_{x,y} = 1 - |\rho_{x,y}|$

where $\rho_{x,y}$is the Pearson correlation between components $x$ and $y$ and ${d_{x,y}}$is the distance between components $x$ and $y$ which generates a square matrix of ${(n. components \cdot n. runs)}$rows and $(n. components \cdot n. runs)$ columns. Exceptionally, for the *AffinityPropagation* clustering algorithm, we multiplied the Pearson distance by -1 to transform dissimilarities into similarities, the required input of this particular algorithm.

We used the following clustering algorithms from the *scikit-learn* and *scikit-learn-extra* libraries that accepted a precomputed dissimilarity matrix as input:

- *sklearn.cluster.AgglomerativeClustering* with *linkage=”average”* and *n_clusters=100*.
- *sklearn.cluster.AffinityPropagation* with default parameters.
- *sklearn.cluster.DBSCAN* with *min_samples=50*.
- *sklearn.cluster.OPTICS* with *min_samples=50*.
- *sklearn_extra.cluster.KMedoids* with *n_clusters=100*.
- *sklearn_extra.cluster.CommonNNClustering* with *min_samples=50*.

We profiled the run time and memory usage of the algorithms and we evaluated their performance based on the distribution of average silhouette scores per cluster through *sklearn.metrics.silhouette_samples* with the default parameters (*metric='euclidean'*), unless stated otherwise. In addition, to evaluate the possible effects of the metric used to calculate the silhouette scores, we used the same function with precomputed Pearson distances instead of the default Euclidean distances. For density-based clustering algorithms -*CommonNNClustering*, *DBSCAN*, *OPTICS*-, we included the -1 cluster in the analyses.

Finally, we also performed a PCA across all ICA runs using the function *sklearn.decomposition.PCA* to visualize how the different clustering algorithms cluster independent components.

**Reproducibility validation of Sastry (2019)**[**^1^**](https://www.zotero.org/google-docs/?p6L7yd) **’s robust ICA pipeline**

To ensure that our implementation of the robust ICA algorithm could reproduce a previously published approach, we adapted Sastry (2019)[^1^](https://www.zotero.org/google-docs/?QT86pd) ’s scripts from their GitHub repository (<https://github.com/SBRG/precise-db>) and ran it using the same parameters of our implementation. Then, we computed the pairwise Pearson correlations and Jaccard distances between the resulting robust independent components and the robust independent components obtained with *robustica*.

## **Inference of components’ signs across multiple ICA runs**

To be able to cluster ICA components across different runs regardless of their sign and order without precomputing the correlation-based dissimilarity matrix, we equipped *robustica* with a subroutine that infers and corrects the signs of the components before the clustering step. After running *FastICA* multiple times, the subroutine sets the first run of ICA as a reference and computes the pairwise Pearson correlation between the reference and every other run. For every pair, we obtain a correlation matrix and, assuming that every component will only have one similar component in another run since ICA returns independent components, we store the sign of the maximum absolute correlation indicating whether the sign has changed with respect to the reference and use it to correct the signs of the components across runs.

## **Module definition**

Throughout this article, we defined gene modules as described in Saelens (2018)[^5^](https://www.zotero.org/google-docs/?MsF8Sq). For every component in the source matrix, we used *fdrtool::fdrtool* to estimate each gene’s false discovery rate (FDR) based on the distribution of weights and applied a cutoff of FDR<0.01 to select the genes belonging to every module.

## **Subjecting robust components to random noise to quantify their robustness**

We measured how weight standard deviation affects the mean weight magnitude and, consequently, the resulting gene modules when using either Pearson or Euclidean distances with sign-corrected components. We used *np.random.normal* to draw 100 samples of weights of robust independent components using their own mean and standard deviation. Then, for each sample, we defined the gene modules as described above and measured how much the sampled modules differed from the original modules using Jaccard similarity with 1 - *sklearn.metrics.pairwise_distances(metric=”jaccard”).* We used the maximum Jaccard similarity to map the pairs of modules to each other. Finally, we saved summary statistics of the mapped pairs.

## **Mapping modules between *robustica* outputs**

We compared how the gene modules resulting from our sign-corrected feature-compressed robust ICA with Euclidean distance metrics differed from the gene modules resulting from using the classical *Icasso* algorithm by measuring pairwise Jaccard similarities using *proxy::sim*, resulting in a square matrix.

We then used module similarities to map the gene modules from one procedure to the other by taking the pair with maximum Jaccard similarity.

## **Exploring LGG expression profiles through robust ICA**

We used *robustica* to dissect the LGG expression profiles into robust independent components using either the original or revisited *Icasso* algorithm with 100 components across 100 ICA runs.

Then, we explored which components in the mixing matrix were associated with *IDH1* and *TP53* mutation status through an unpaired Wilcoxon Rank Sum test (*stats::wilcox.test*); with sample histological type (*stats::kruskal.test*); with the mitotic index through Spearman correlation (*stats::cor*); and with patient overall survival through a Cox Proportional-Hazards regression (*survival::coxph*).

Finally, we further characterized the gene signatures in the source matrix that drive these associations in components 72 and 12, as examples of components identified by either both or only our version of the *Icasso* algorithm. We defined a gene set by taking the genes with extreme weights in that component as explained above and performed a gene set enrichment analysis for GO biological processes and MSigDB’s hallmark signatures (*clusterProfiler::enricher*) using the default parameters.

## **Software**

| **Name** | **Version** | **Reference** | **Repository** |
| --- | --- | --- | --- |
| Python | 3.8.3 | Van Rossum (2009)[^6^](https://www.zotero.org/google-docs/?2SMGrd) | <https://github.com/python/cpython> |
| robustica | 0.1.2 | (this publication) | <https://github.com/CRG-CNAG/robustica> |
| snakemake | 5.31.1 | Mölder (2021)[^7^](https://www.zotero.org/google-docs/?QiqBAX) | <https://github.com/snakemake/snakemake> |
| rpy2 | 3.3.6 | - | <https://github.com/rpy2/rpy2> |
| pandas | 1.1.2 | McKinney (2010)[^8^](https://www.zotero.org/google-docs/?1jgRDq) | <https://github.com/pandas-dev/pandas> |
| numpy | 1.19.2 | Harris (2020)[^9^](https://www.zotero.org/google-docs/?mrdFLb) | <https://github.com/numpy/numpy> |
| matplotlib | 3.3.1 | Hunter (2007)[^10^](https://www.zotero.org/google-docs/?SINo4z) | <https://github.com/matplotlib/matplotlib> |
| seaborn | 0.10.1 | Waskom (2021)[^11^](https://www.zotero.org/google-docs/?ZQKkop) | <https://github.com/mwaskom/seaborn> |
| scikit-learn | 0.23.2 | Pedregosa (2011)[^12^](https://www.zotero.org/google-docs/?0BHGb0) | <https://github.com/scikit-learn/scikit-learn> |
| scikit-learn-extra | 0.2.0 | - | <https://github.com/scikit-learn-contrib/scikit-learn-extra> |
| scipy | 1.7.1 | Virtanen (2020)[^13^](https://www.zotero.org/google-docs/?UzlndH) | <https://github.com/scipy/scipy> |
| memory_profiler | 0.58.0 | - | <https://github.com/pythonprofilers/memory_profiler> |
| tqdm | 4.64.0 | - | <https://github.com/tqdm/tqdm> |
| R | 4.0.3 | R Core Team (2020)[^14^](https://www.zotero.org/google-docs/?ipcugz) | <https://github.com/wch/r-source> |
| circlize |  | Gu (2014)[^15^](https://www.zotero.org/google-docs/?vEPRJ5) | <https://github.com/jokergoo/circlize> |
| clusterProfiler | 3.99.0 | - | <https://github.com/YuLab-SMU/clusterProfiler> |
| ComplexHeatmap |  | Gu (2016)[^16^](https://www.zotero.org/google-docs/?ZU31vB) | <https://github.com/jokergoo/ComplexHeatmap> |
| cowplot |  | - | <https://github.com/wilkelab/cowplot> |
| extrafont | 0.17 | - | <https://github.com/wch/extrafont> |
| frdtool | 1.2.16 | Strimmer (2008)[^17^](https://www.zotero.org/google-docs/?oeICOd) | <http://www.strimmerlab.org/software/fdrtool/> |
| ggplotify |  | - | <https://github.com/GuangchuangYu/ggplotify> |
| ggpubr | 0.4.0 | - | <https://github.com/kassambara/ggpubr> |
| ggrepel | 0.8.2 | - | <https://github.com/slowkow/ggrepel> |
| gridExtra |  | - | <https://github.com/baptiste/gridextra> |
| proxy | 0.4.25 | - | <https://github.com/cran/proxy> |
| scattermore |  | - | <https://github.com/exaexa/scattermore> |
| survival | 3.2.7 | - | <https://github.com/therneau/survival> |
| survminer | 0.4.8 | - | <https://github.com/kassambara/survminer> |
| tidyverse | 1.3.0 | Wickham (2019)[^18^](https://www.zotero.org/google-docs/?huCYIV) | <https://github.com/tidyverse/tidyverse> |

#

# **SUPPLEMENTARY FIGURES**

**
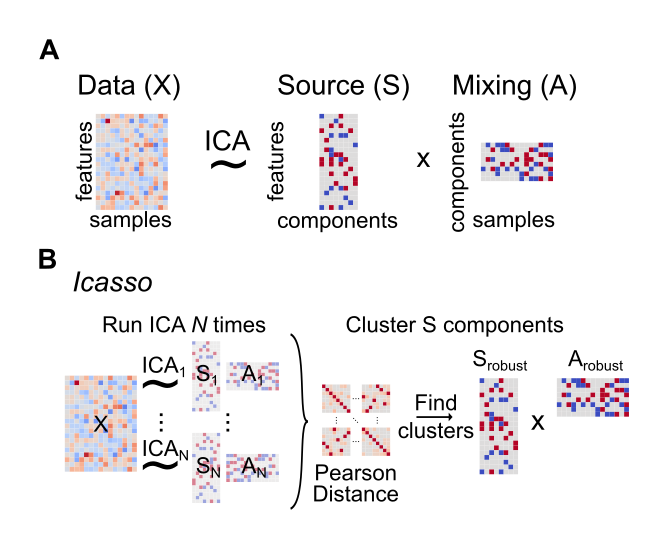
**

### **Supplementary Figure 1. Independent Component Analysis (ICA) and robust ICA with the *Icasso* algorithm.**

(**A**) ICA is a matrix factorization algorithm applied for blind-source separation problems that decompose a data matrix (*X*) into *n* independent components generating a source matrix (*S*) and a mixing matrix (*A*) with information on how features and samples contribute to each independent component, respectively. (**B**) The *Icasso* algorithm overcomes the inherent randomness of the *FastICA* algorithm -a widespread algorithm to perform ICA- by running ICA multiple times and clustering the resulting independent components in *S* across all runs using an agglomerative clustering approach with average linkage and a Pearson distance matrix ${(n. components \cdot n. runs)}^{2}$dimensions as input.


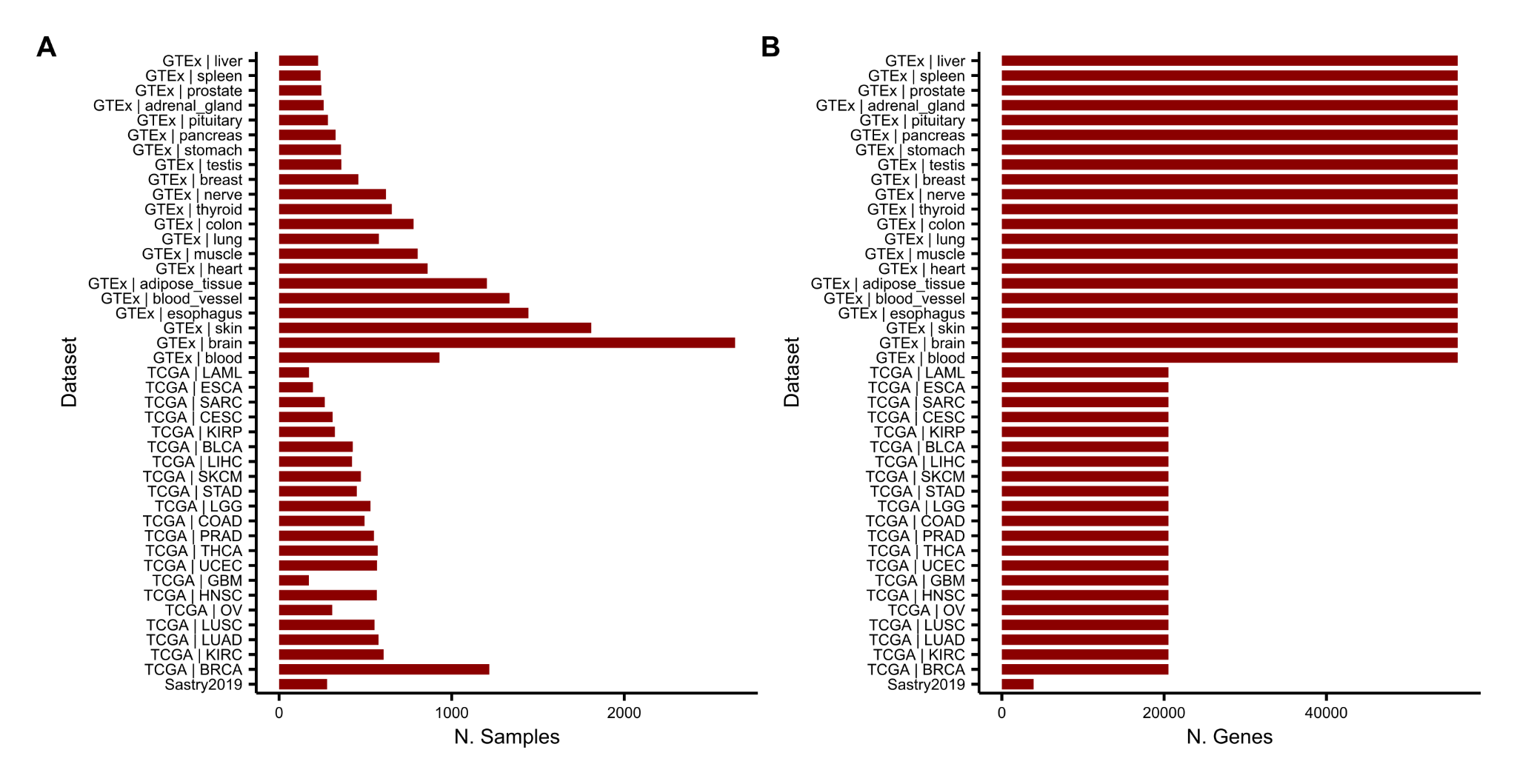


### **Supplementary Figure 2. Overview of the transcriptomic datasets considered.**

### Bar plots of the number of (**A**) samples and (**B**) genes (x-axis) in each of the 43 datasets (y-axis) used to benchmark the clustering step of the robust ICA algorithm. We considered datasets from the GTEx and TCGA projects focused on human samples, and Study abbreviations for TCGA datasets can be found online at <https://gdc.cancer.gov/resources-tcga-users/tcga-code-tables/tcga-study-abbreviations>.

###

###
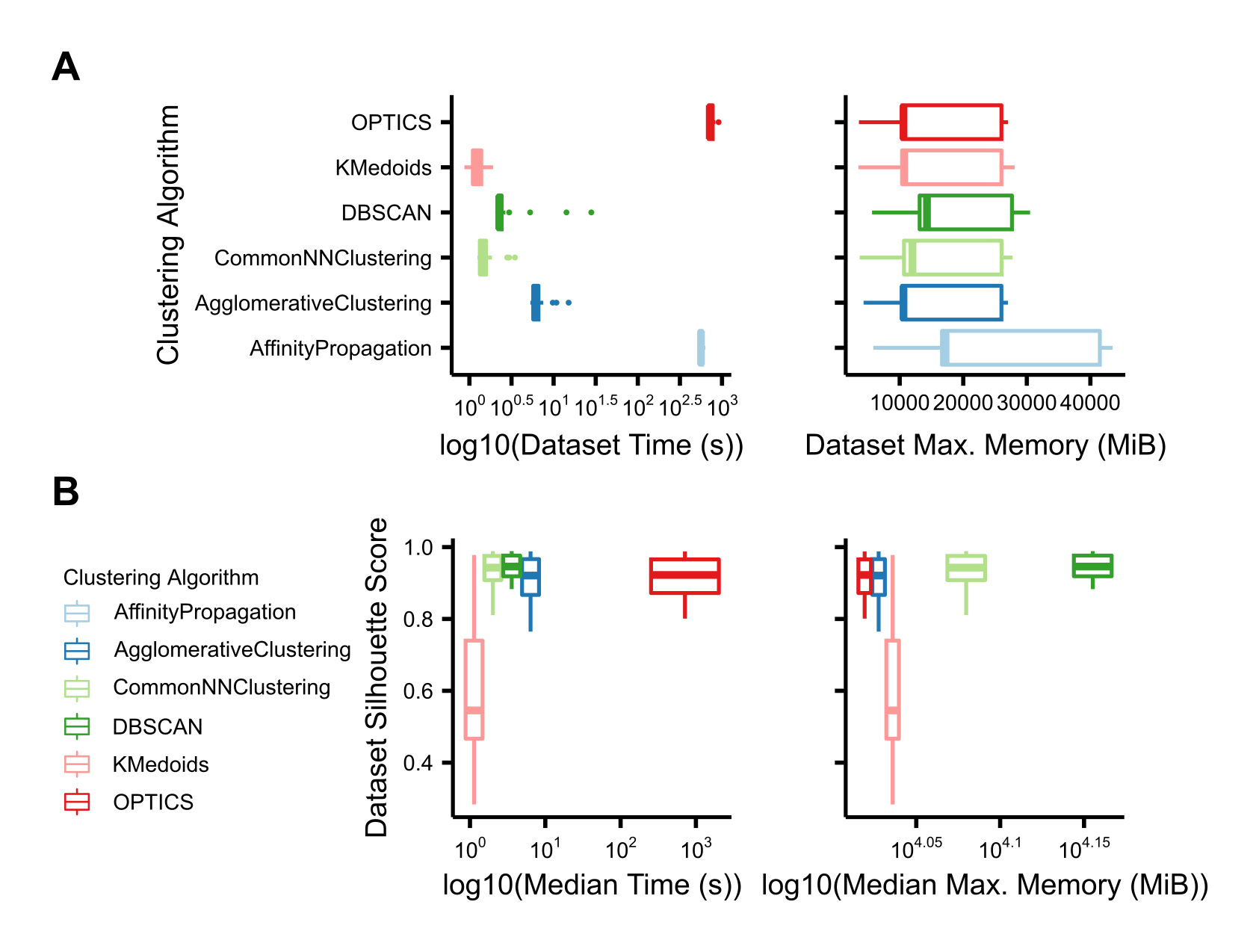


### **Supplementary Figure 3. *DBSCAN* shows the best performance to compute robust independent components.**

(**A**) Distributions of time (seconds) and maximum memory usage (MiB) required to cluster the independent components produced across 100 runs of ICA with *n_components=100* (x-axis) for the 43 different datasets using 6 different clustering algorithms (y-axis). (**B**) Median time (seconds) and median maximum memory usage (MiB) (x-axis) compared to the median silhouette score (y-axis) for the robust independent components computed with 6 different clustering algorithms and dissecting the 43 different datasets. Silhouette scores range from -1 to 1, 1 indicating the best clustering goodness.


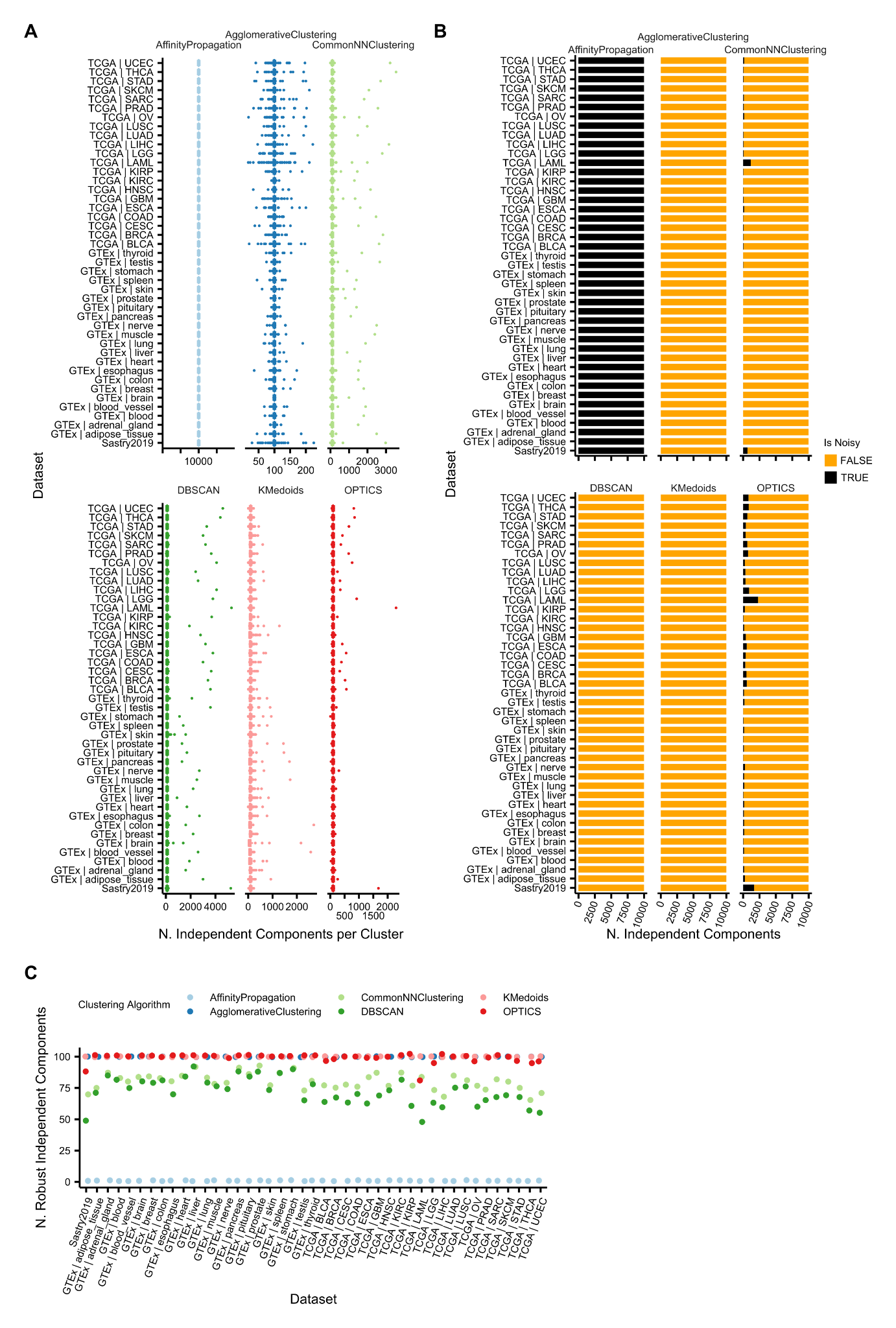


### **Supplementary Figure 4. Clustering algorithms evaluation.**

(**A**) The number of independent components across 100 runs of ICA with *n_components=100* that each clustering algorithm placed into a cluster (x-axis) for the 43 different datasets (y-axis). (**B**) The number of independent components across 100 runs of ICA with *n_components=100* that were considered “noise” (i.e. placed into cluster “-1”) by the density-based clustering algorithms (x-axis) for the 43 different datasets (y-axis). (**C**) The number of robust independent components resulting from the 6 different clustering algorithms (y-axis) across the 43 different datasets (x-axis).


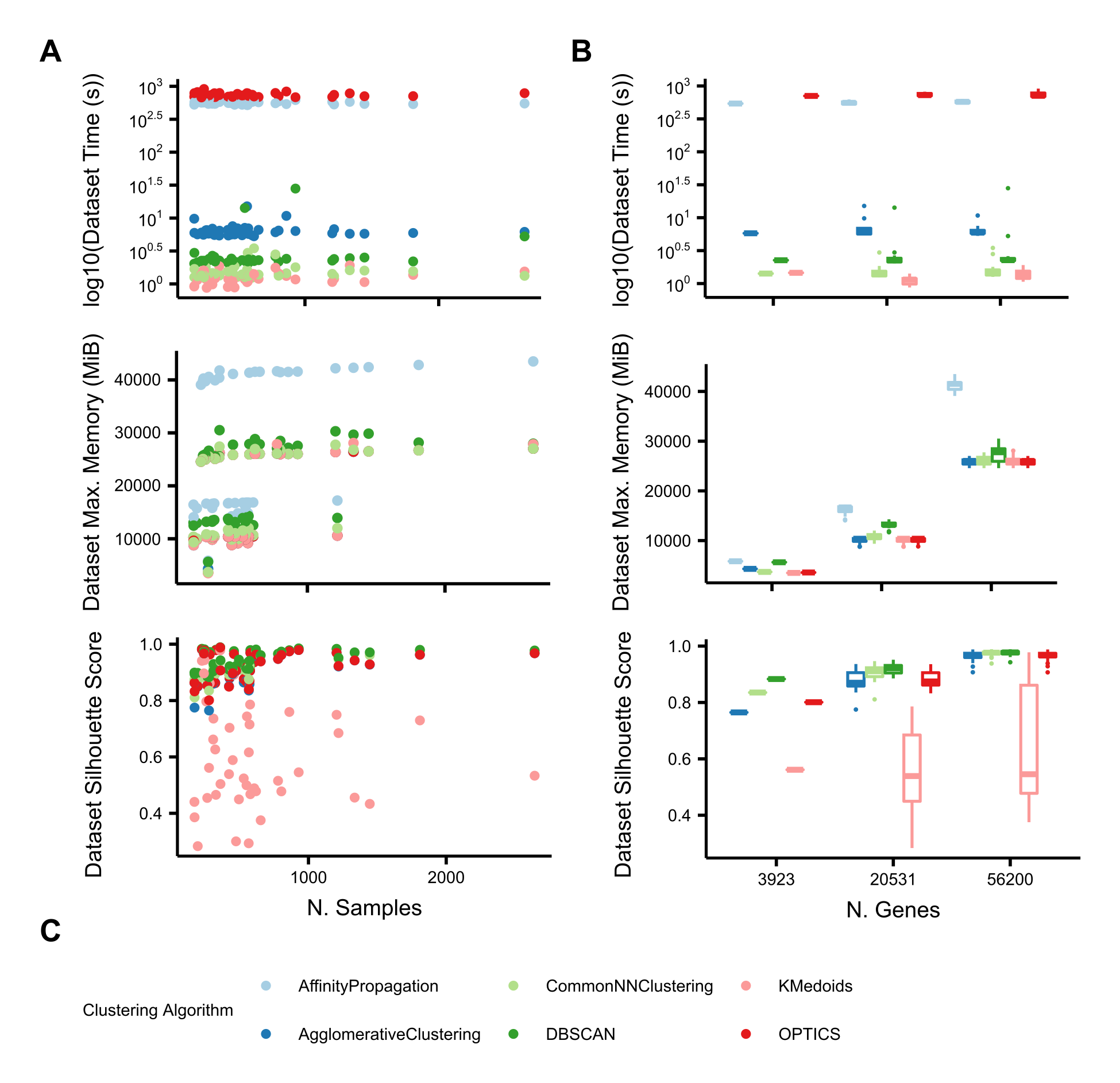


### **Supplementary Figure 5. Evaluation of the scalability of the different clustering algorithms.**

Median time, memory, and silhouette scores (y-axis) measured for clustering the 43 different datasets considering either (**A**) their number of samples (x-axis), or (**B**) their number of genes (x-axis). Note that the actual input of the clustering algorithms consists of the 10,000 independent components produced across 100 runs of ICA with *n_components=100*. (**C**) Common color codes for the 6 clustering algorithms depicted in the rest of the figure panels.


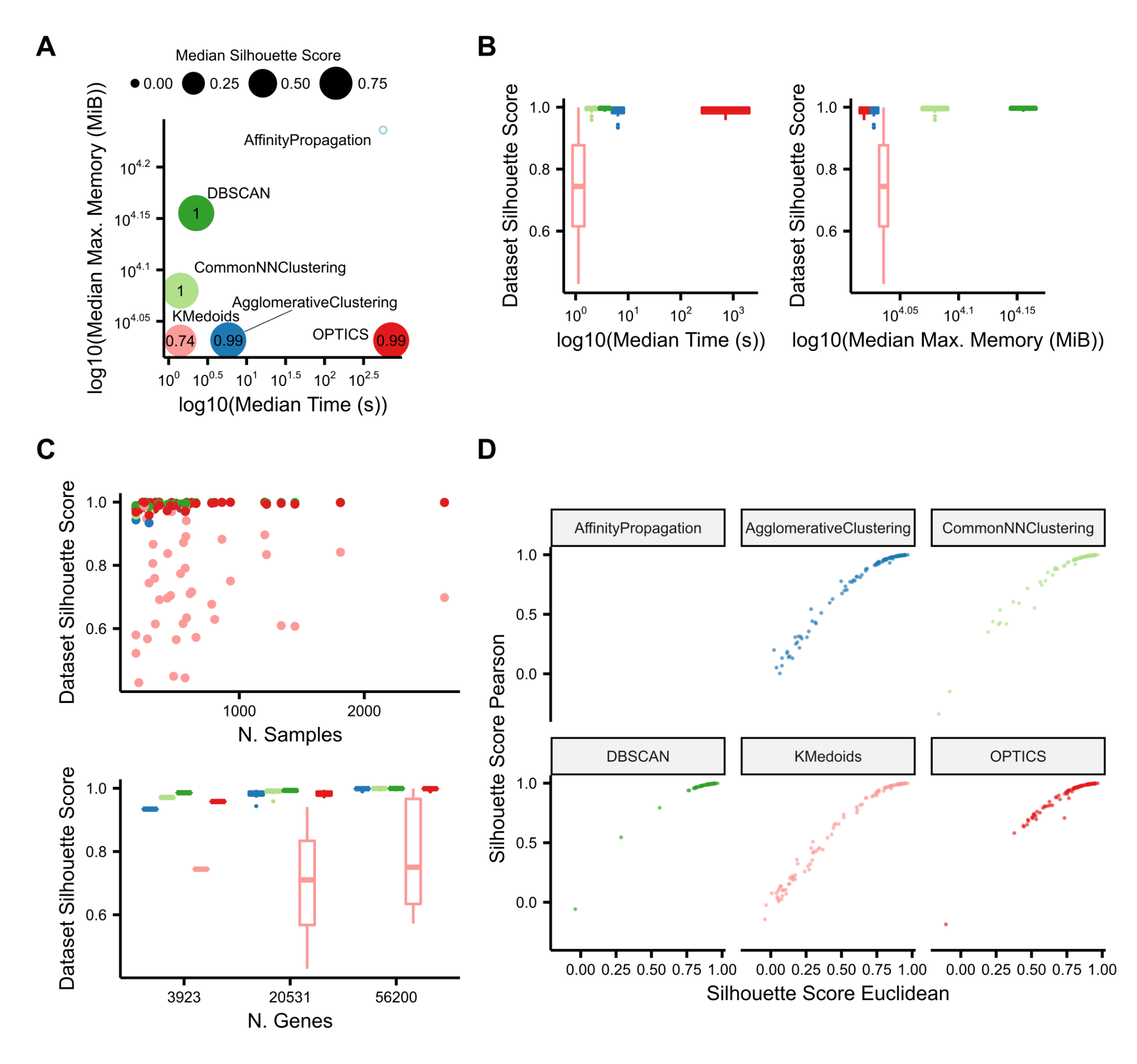


### **Supplementary Figure 6. Default Euclidean-based silhouette scores show higher resolution than Pearson-based silhouette scores to benchmark the different clustering algorithms.**

(**A**) Clustering algorithms comparative for 43 different transcriptomic datasets. Median time (x-axis) and the median maximum memory usage (y-axis) for each clustering algorithm to cluster 100 ICA runs with 100 components each. Dot sizes indicate median Pearson distance-based silhouette scores. Silhouette scores range from -1 to 1, 1 indicating the best clustering goodness. Note that the *AffinityPropagation* algorithm is represented as an empty dot due to silhouette scores being non-computable as convergence was not reached with the tested parameters. (**B**) Median time (seconds) and median maximum memory usage (MiB) (x-axis) compared to the median Pearson distance-based silhouette score (y-axis) for the robust independent components computed with 6 different clustering algorithms and dissecting the 43 different datasets. (**C**) Median Pearson distance-based silhouette scores (y-axis) measured for clustering the 43 different datasets considering either (top) their number of samples (x-axis), or (bottom) their number of genes (x-axis). Note that the actual input of the clustering algorithms consists of the 10,000 independent components produced across 100 runs of ICA with *n_components=100*. (**D**) Comparison of cluster average silhouette scores produced using either default Euclidean distance (x-axis) or Pearson distance (y-axis) as the metric for the evaluation of the different clustering algorithms by dissecting Sastry (2019)[^1^](https://www.zotero.org/google-docs/?3lIKDW) ’s dataset. Panels B and C follow the same color codes indicated in A and D.

**
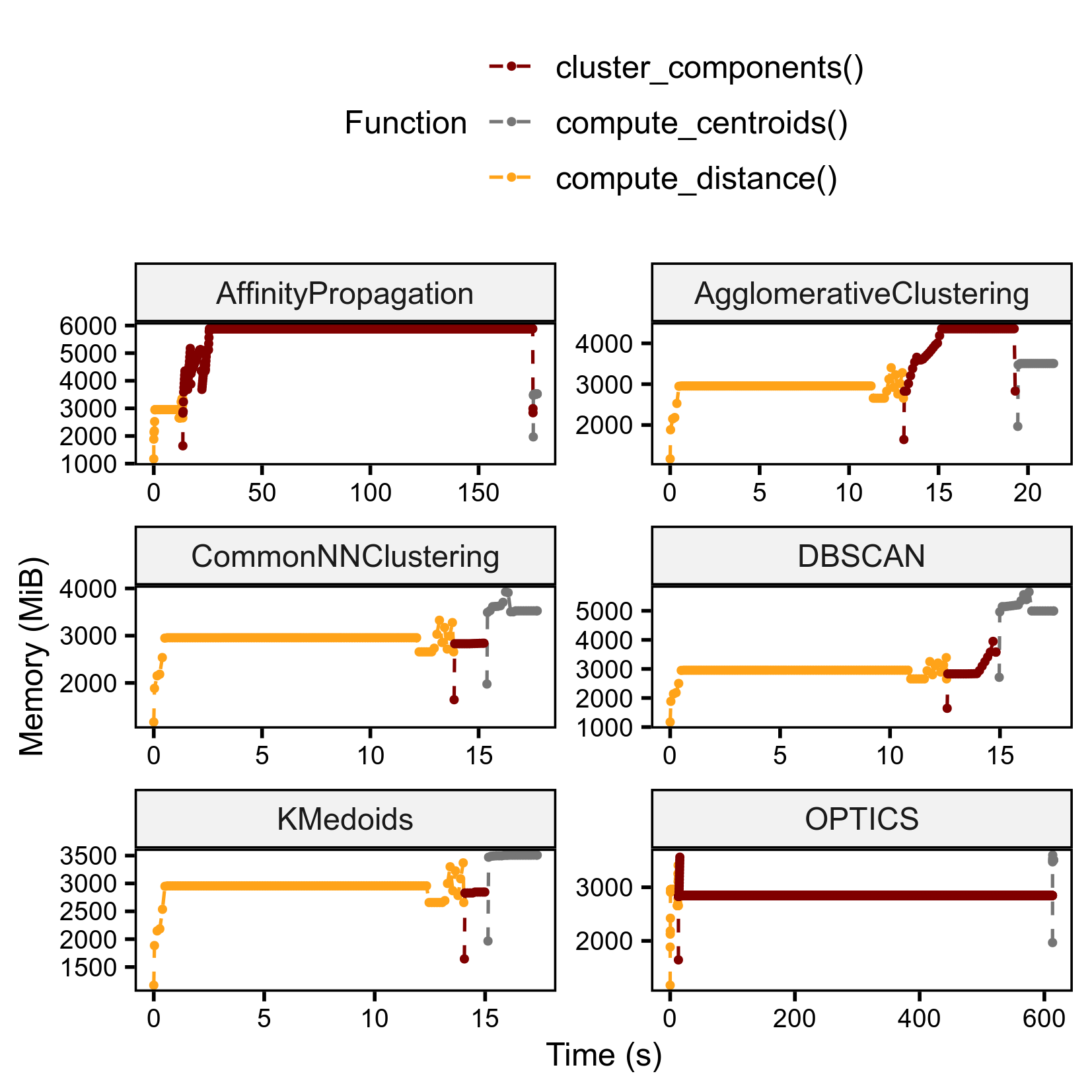
**

### **Supplementary Figure 7. Computing the Pearson distance matrix takes the most time using the *DBSCAN* algorithm.**

Memory usage (y-axis) across time (x-axis) and substeps (functions) to compute robust independent components in our comparison of clustering algorithms by dissecting Sastry (2019)[^1^](https://www.zotero.org/google-docs/?qbwtiK) ’s dataset.


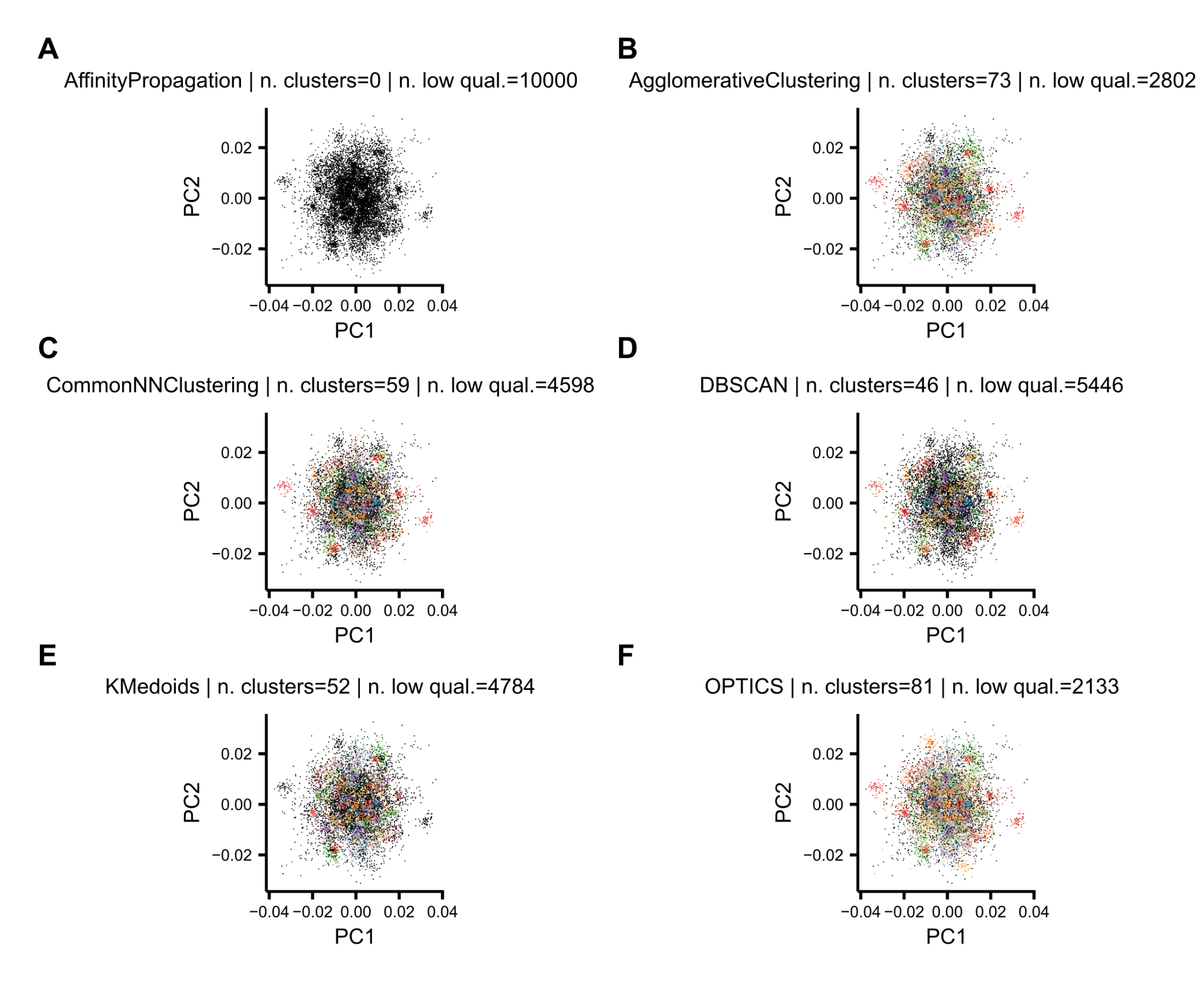


### **Supplementary Figure 8. Illustration of the clustering decision across the 6 different clustering algorithms.**

Scatterplots for the two first principal components computed from all 10,000 ICA runs (100 runs of ICA with *n_components=100*) and colored each independent component with the corresponding cluster label using Sastry (2019)[^1^](https://www.zotero.org/google-docs/?3OOOPL) ’s dataset (**A**-**F**). In this example, we highlighted in black those independent components that belong to clusters with an average silhouette score lower than 0.5, an indicator that the corresponding robust independent component will not be as stable as robust independent components of higher quality. We can appreciate how points within highly crowded regions are clustered together in density-based algorithms leaving out unstable independent components likely originating from ICA’s inherent randomness. In fact, *DBSCAN* is the algorithm with the highest number of points within low-quality clusters, which may lead to precise robust independent components calculated with at least 50 components in this benchmark.

### **
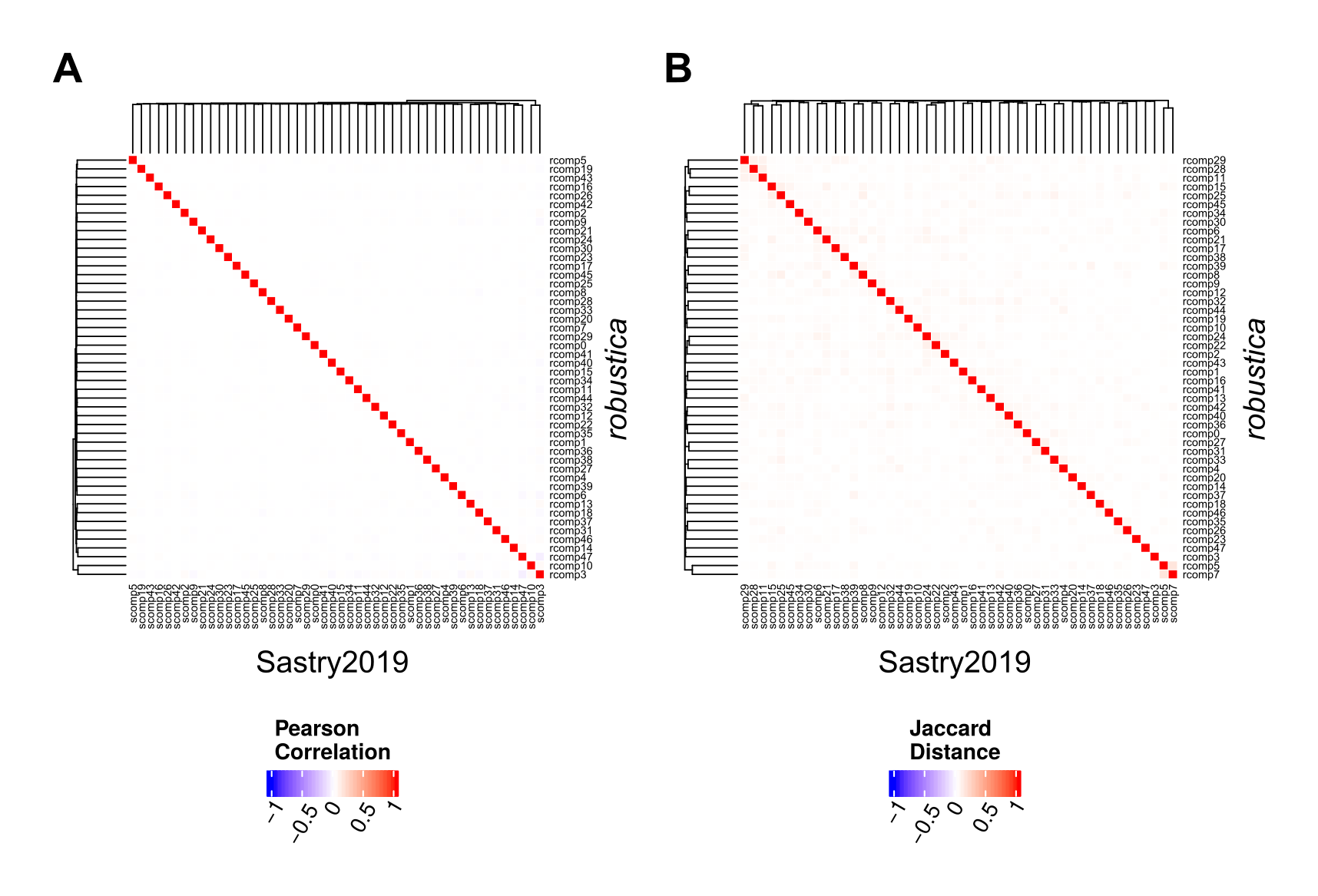
**

### **Supplementary Figure 9. *robustica* can reproduce the robust independent components found with Sastry (2019)’**[**^1^**](https://www.zotero.org/google-docs/?lwrdNC) **s clustering algorithm.**

### Compared to our results, Sastry (2019)[^1^](https://www.zotero.org/google-docs/?IsgPpw) identified more robust independent components (92 in total) performing robust ICA with *DBSCAN* using different clustering parameters. We confirmed the source of this difference by running Sastry’s (2019)[^1^](https://www.zotero.org/google-docs/?cjyVmb) ’s clustering algorithm with the same parameters that we used in the present analysis. As expected, this resulted in 46 highly similar robust independent components considering either pairwise Pearson correlations (**A**) or Jaccard distances (**B**).


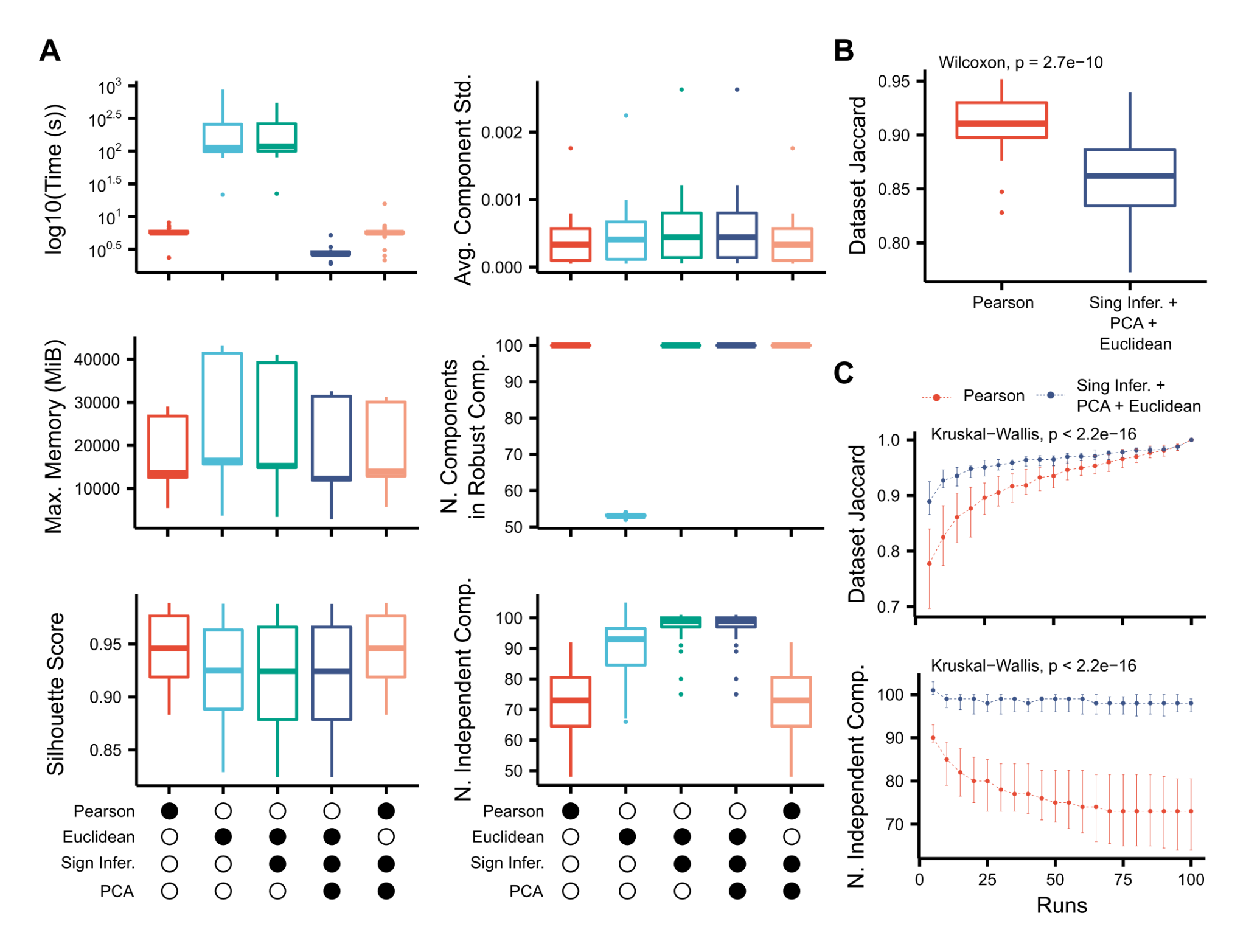


### **Supplementary Figure 10. Our sign inference-and-correction subroutine enables using Euclidean distances for clustering and results in highly stable robust independent components.**

### (**A**) Distributions of performance metrics (y-axis) for different variants of the *Icasso* algorithm (x-axis). Each panel illustrates how our versions of the *Icasso* algorithm perform at dissecting the 43 datasets using: the original Pearson distance matrix (Pearson); plain Euclidean distance (Euclidean); Euclidean distance after our sign inference-and-correction subroutine (Sign Infer. + Euclidean); Euclidean distance after our sign inference-and-correction subroutine and feature compression with PCA (Sign Infer. + PCA + Euclidean); or Pearson distance after our sign inference-and-correction and feature compression with PCA (Sign Infer. + PCA + Pearson). For each dataset and algorithm version, we measured its: time; memory; median of averaged silhouette scores of each robust independent component; the median of averaged standard deviation of the weights of each robust independent component; the median number of independent components in each robust component; and the final number of robust independent components. (**B**) Median of averaged Jaccard similarities (y-axis) between the gene modules defined using inferred robust independent components and their corresponding weights subjected to random noise sampled 100 times across the 43 datasets for the original and the revisited *Icasso* algorithms (x-axis). (**C**) Distributions (mean and standard deviation) of median maximum Jaccard similarities (up; y-axis) and the number of robust independent components (down; y-axis) between the gene modules defined using a different number of ICA runs (x-axis) compared with the gene modules defined using 100 ICA runs for both the original and the revisited approaches across the 43 datasets.

###

### **
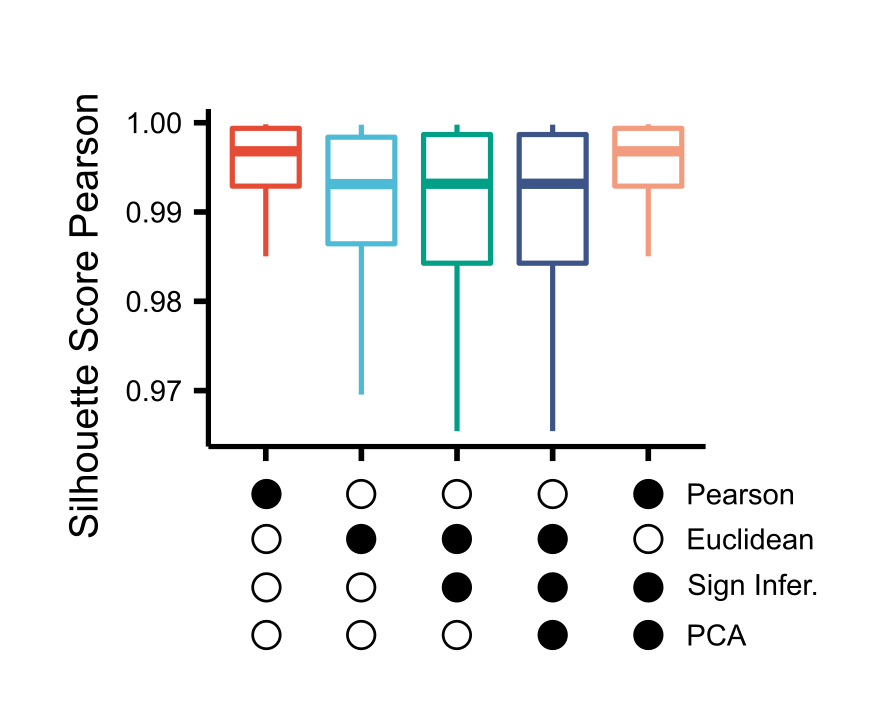
**

### **Supplementary Figure 11. Evaluation of our sign inference-and-correction subroutine using Pearson distance-based silhouette scores.**

### Distributions of the median of averaged Pearson-based silhouette scores (y-axis) for different variants of the Icasso algorithm (x-axis) illustrating how our versions of the Icasso algorithm perform at dissecting the 43 datasets using: the original Pearson distance matrix (Pearson); plain Euclidean distance (Euclidean); Euclidean distance after our sign inference-and-correction subroutine (Sign Infer. + Euclidean); Euclidean distance after our sign inference-and-correction subroutine and feature compression with PCA (Sign Infer. + PCA + Euclidean); or Pearson distance after our sign inference-and-correction and feature compression with PCA (Sign Infer. + PCA + Pearson).

### **
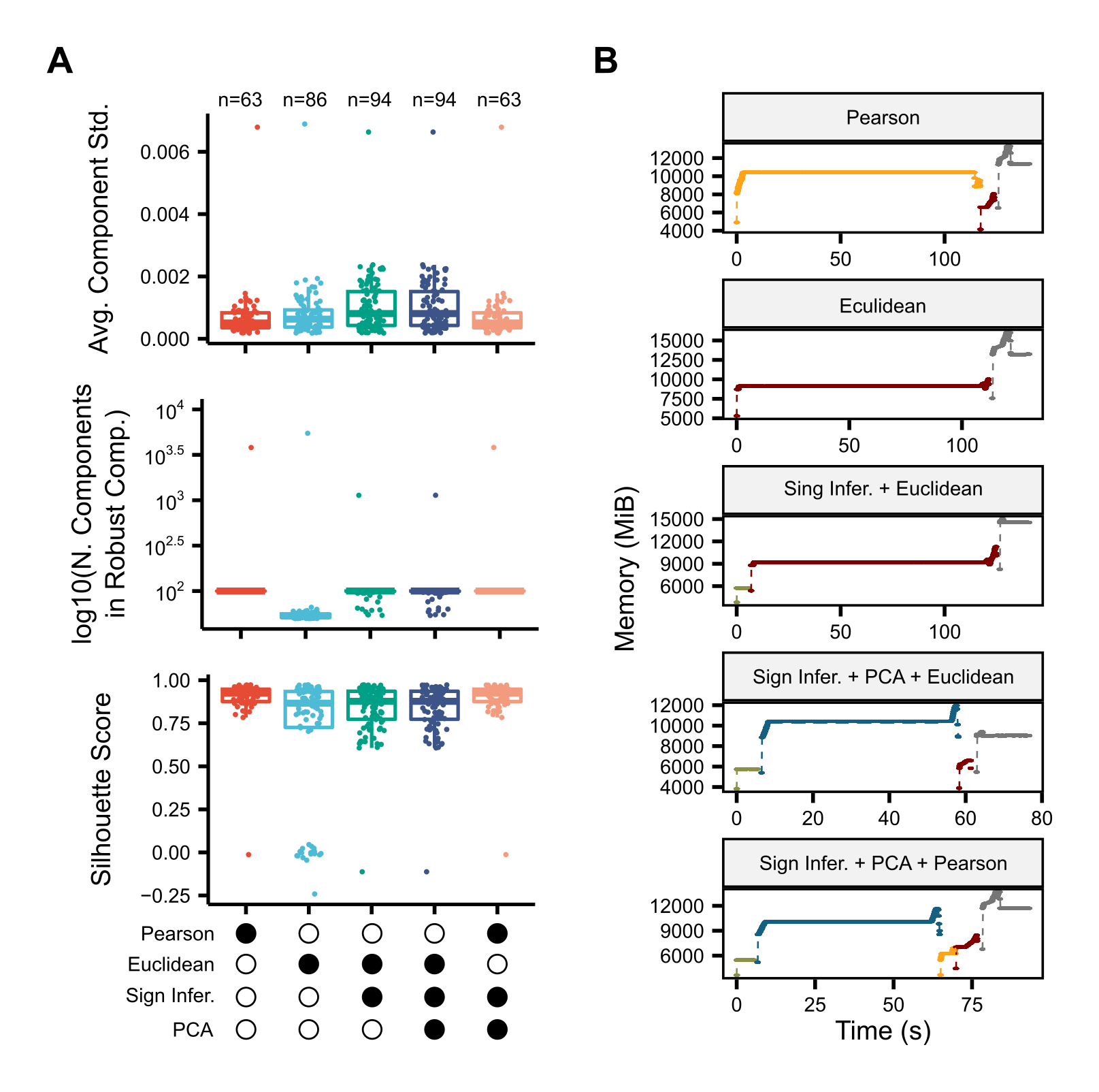
**

### **Supplementary Figure 12. Evaluation of our sign inference-and-correction subroutine in the LGG dataset.**

(**A**) For different versions of the *Icasso* algorithm (x-axis), we obtained the distributions (y-axis) of the (up) average standard deviations of the weights of robust independent components; (middle) the number of independent components placed in the same robust independent component; (down) the average silhouette score of each robust independent component. (**B**) Memory usage (y-axis) across time (x-axis) and substeps of every version of the *Icasso* algorithm: computing Pearson distance (yellow); clustering components with *DBSCAN* (dark red); computing centroids (grey); inferring components’ signs (dark green); compressing feature space with PCA (dark blue).

###

### **
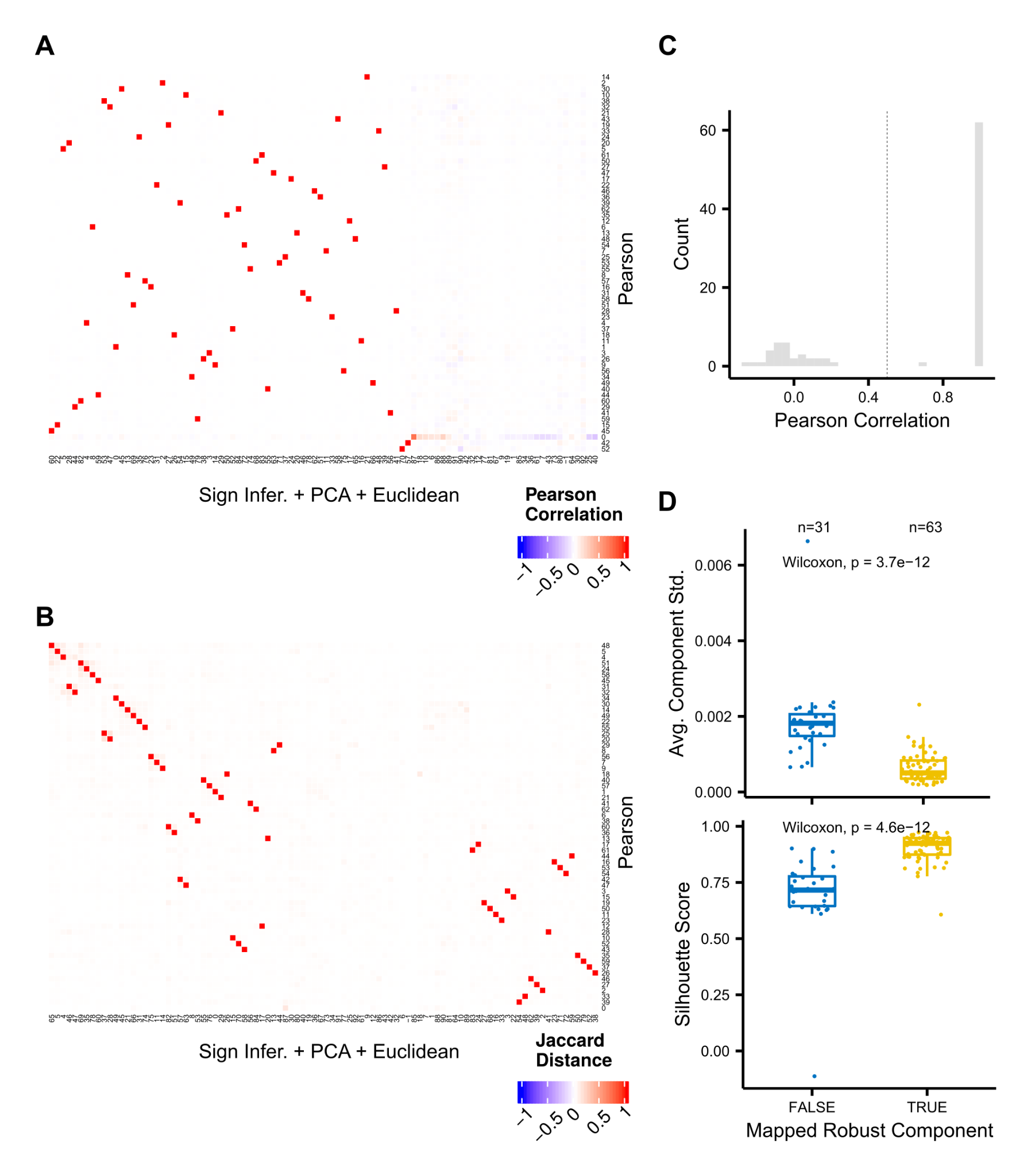
**

### **Supplementary Figure 13. Comparison of components inferred with different *Icasso* versions using the LGG dataset.**

Heatmaps illustrating pairwise (**A**) Pearson correlations and Jaccard distances (**B**) between robust independent components produced by the original (*Pearson*) (y-axis) or revisited (*Sign Infer. + PCA + Euclidean*) (x-axis) versions of the *Icasso* algorithms. (**C**) Histogram of Pearson correlations (x-axis) used to map robust independent components found through the different versions of the *Icasso* algorithm. Components with a maximum pairwise Pearson correlation lower than 0.5 (vertical dashed line) were considered as not mapped. (**D**) Distributions of (up) the average standard deviation of the weights of robust independent components and (down) their average silhouette score (y-axis) for mapped and unmapped robust independent components in our revisited version of the *Icasso* algorithm (x-axis).

**
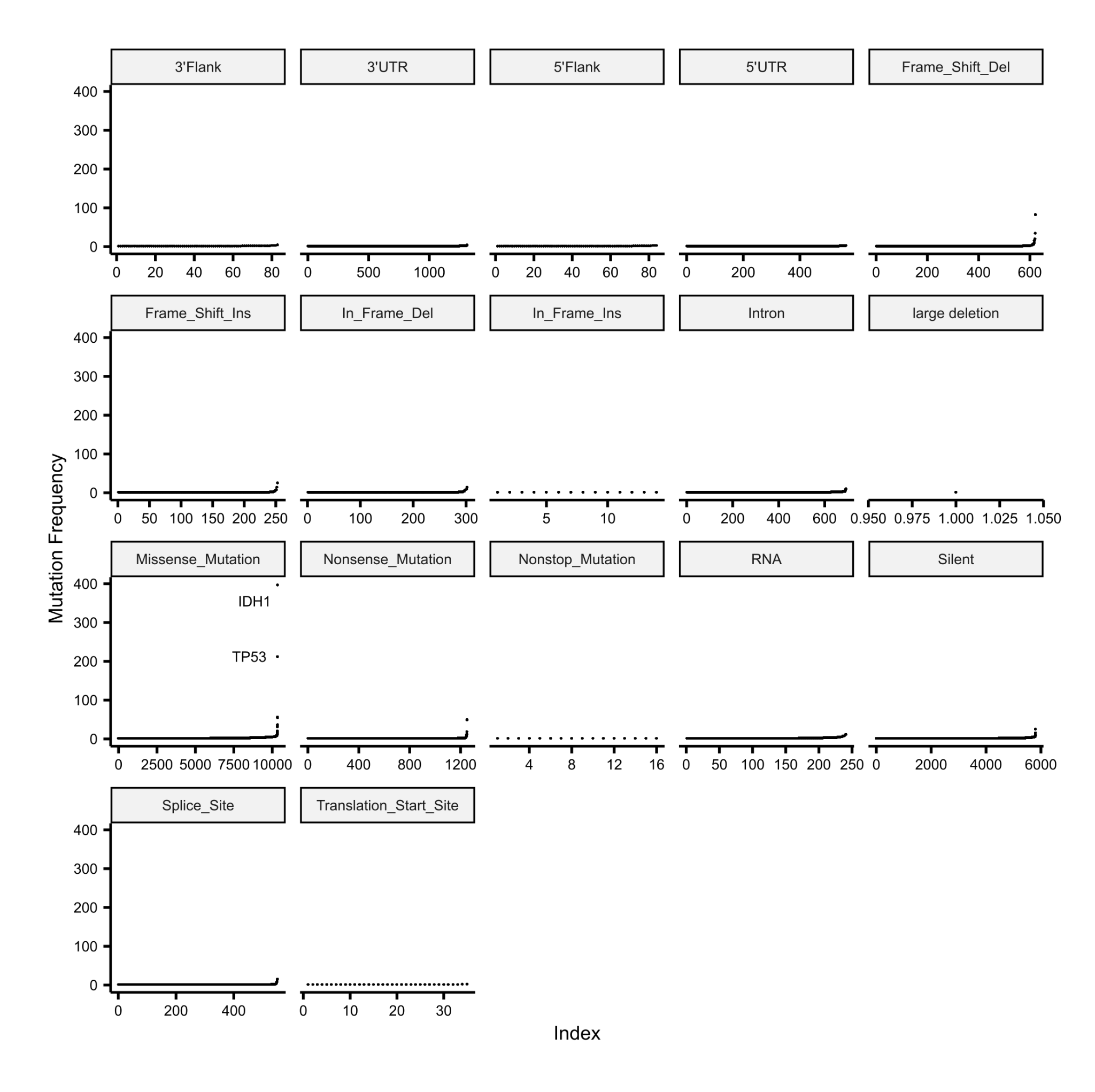
**

### **Supplementary Figure 14. Gene mutation frequency in LGG patients.**

Number of mutations per gene (y-axis) measured across 530 LGG patients considered arranged in increasing order (x-axis) and classified according to their predicted effect (panel label).

###

### **
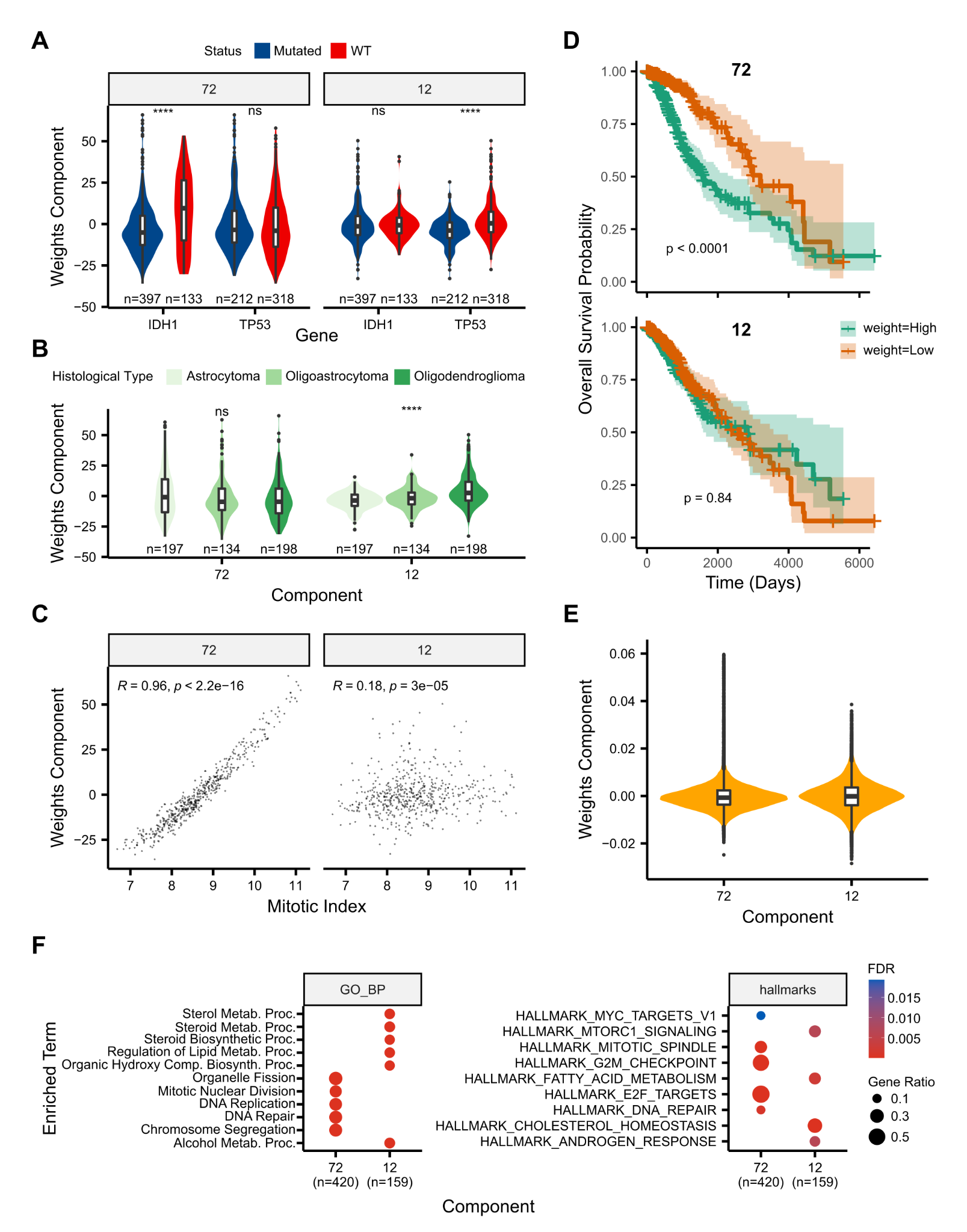
**

### **Supplementary Figure 15. *robustica* identifies disease-relevant gene modules.**

### (**A**) Distribution of sample weights in components 72 and 12 in the robust mixing matrix (y-axis) among patients with (Mutated) or without (WT) mutations (x-axis) in genes *IDH1* and *TP53* (panel labels)*.* We used a Wilcoxon Rank Sum test to assess the statistical differences between the groups (p-values: <0.0001 (****), >0.05 (ns)). (**B**) Distribution of sample weights in components 72 and 12 in the robust mixing matrix (y-axis) among patients with different histological types of tumor (Astrocytoma, Oligoastrocytoma, or Oligodendroglioma) (x-axis)*.* We used a Kruskal Wallis test to assess the statistical differences between the groups (p-values: <0.0001 (****), >0.05 (ns)). (**C**) Relationship between weights in component 7 in the robust mixing matrix (y-axis) and each sample’s mitotic index (x-axis). The Spearman correlation coefficient and its corresponding p-value are indicated at the top left (*R* and *p*, respectively). (**D**) Kaplan-Meier curves of patients with weights higher or lower than the median weight in components (up) 72 or (down) 12 in the robust mixing matrix. The p-value of the log-rank test between the two groups is indicated at the bottom left. (**E**) Distribution of weights (y-axis) in components 72 and 12 in the robust source matrix (x-axis) used to define the gene module corresponding to our sample features of interest. (**F**) Results from overrepresentation tests of our gene module defined from components 72 and 12 (x-axis) and the (left) Gene Ontology (GO) Biological Processes or (right) MSigDB Hallmarks databases (y-axis).

#

# **SUPPLEMENTARY TABLES**

### Supplementary tables were made available through the Zenodo online repository: <https://zenodo.org/record/6833937>.

### **Overview of datasets analyzed**

#### Supplementary Table 1. Overview of datasets used in this study.

### **Benchmark clustering algorithms**

#### Supplementary Table 2. Memory usage profiles across time for each subroutine of the clustering step for each of the algorithms and datasets considered.

#### Supplementary Table 3. The clustering evaluation for each of the components for each of the algorithms and datasets considered.

#### Supplementary Table 4. Summary statistics of the clustering evaluation for each of the components for each of the algorithms and datasets considered.

#### Supplementary Table 5. PCA across the 10,000 independent components produced upon running ICA 100 times with *n_components=100* using Sastry (2019)[^1^](https://www.zotero.org/google-docs/?I48JgV) ’s transcriptomic profiles.

#### Supplementary Table 6. Source matrix weight means using Sastry (2019)[^1^](https://www.zotero.org/google-docs/?zpZRIe) ’s transcriptomic profiles obtained with *robustica*.

#### Supplementary Table 7. Source matrix weight means using Sastry (2019)[^1^](https://www.zotero.org/google-docs/?4IqlNH) ’s transcriptomic profiles obtained with Sastry (2019)[^1^](https://www.zotero.org/google-docs/?97dcJ0) ’s clustering step adapted to the parameters used in this study.

### **Benchmark sign inference-and-correction subroutine**

#### Supplementary Table 8. The clustering evaluation for each of the components for each of the clustering metrics and datasets considered. We evaluated the following metric variants: Pearson distances (*icasso*), Euclidean distances (*robustica_nosign*), our subroutine for component sign inference-and-correction together with Euclidean distances (*robustica*); our subroutine for component sign inference-and-correction and compression of the feature space with PCA together with Euclidean distances (*robustica_pca*); and our subroutine for component sign inference-and-correction and compression of the feature space with PCA together with Pearson distances (*icasso_pca*).

#### Supplementary Table 9. Summary statistics of the clustering evaluation for each of the components for each of the clustering metrics and datasets considered. We evaluated the following metric variants: Pearson distances (*icasso*), Euclidean distances (*robustica_nosign*), our subroutine for component sign inference-and-correction together with Euclidean distances (*robustica*); our subroutine for component sign inference-and-correction and compression of the feature space with PCA together with Euclidean distances (*robustica_pca*); and our subroutine for component sign inference-and-correction and compression of the feature space with PCA together with Pearson distances (*icasso_pca*).

#### Supplementary Table 10. Evaluation of gene module robustness upon subjecting robust independent components estimated with the different versions of the Icasso algorithm to random noise. We evaluated the following metric variants: Pearson distances (*icasso*), Euclidean distances (*robustica_nosign*), our subroutine for component sign inference-and-correction together with Euclidean distances (*robustica*); our subroutine for component sign inference-and-correction and compression of the feature space with PCA together with Euclidean distances (*robustica_pca*); and our subroutine for component sign inference-and-correction and compression of the feature space with PCA together with Pearson distances (*icasso_pca*).

#### Supplementary Table 11. Evaluation of gene module reproducibility using a different number of ICA runs to compute robust independent components for each version of the algorithm. We evaluated the following metric variants: Pearson distances (*icasso*), Euclidean distances (*robustica_nosign*), our subroutine for component sign inference-and-correction together with Euclidean distances (*robustica*); our subroutine for component sign inference-and-correction and compression of the feature space with PCA together with Euclidean distances (*robustica_pca*); and our subroutine for component sign inference-and-correction and compression of the feature space with PCA together with Pearson distances (*icasso_pca*).

### **Case study**

#### Supplementary Table 12. Memory usage profiles across time for each subroutine of the clustering step for each of the clustering metrics using LGG transcriptomic profiles.

#### Supplementary Table 13. Source matrix weight means using LGG transcriptomic profiles obtained with Pearson distances.

#### Supplementary Table 14. Source matrix weight standard deviations using LGG transcriptomic profiles obtained with Pearson distances.

#### Supplementary Table 15. Source matrix weight means using LGG transcriptomic profiles obtained with our subroutine for component sign inference-and-correction and compression of the feature space with PCA together with Euclidean distances.

#### Supplementary Table 16. Source matrix weight standard deviations using LGG transcriptomic profiles obtained with our subroutine for component sign inference-and-correction and compression of the feature space with PCA together with Euclidean distances.

#### Supplementary Table 17. Mixing matrix weight means using LGG transcriptomic profiles obtained with our subroutine for component sign inference-and-correction and compression of the feature space with PCA together with Euclidean distances.

#### Supplementary Table 18. Overlap evaluation of the LGG robust independent components between the classical (*Pearson*) and revisited (*Sign Infer. + PCA + Euclidean*) *Icasso* algorithm.

#### Supplementary Table 19. Somatic mutations detected in the samples of the LGG dataset.

#### Supplementary Table 20. Sample metadata of the LGG dataset.

#### Supplementary Table 21. Statistical associations between sample metadata and mixing matrix weights (Sup. Tab. 17) of the LGG dataset.

#### Supplementary Table 22. Gene modules obtained from source matrix weights (Sup. Tab. 16).

#### Supplementary Table 23. Gene set overlap analysis results for gene modules (Sup. Tab. 22).

# **REFERENCES**

[1. Sastry, A. V. *et al.* The Escherichia coli transcriptome mostly consists of independently regulated modules. *Nat. Commun.* **10**, 5536 (2019).](https://www.zotero.org/google-docs/?4Us6eu)

[2. Goldman, M. J. *et al.* Visualizing and interpreting cancer genomics data via the Xena platform. *Nat. Biotechnol.* **38**, 675–678 (2020).](https://www.zotero.org/google-docs/?4Us6eu)

[3. Yang, Z. *et al.* Correlation of an epigenetic mitotic clock with cancer risk. *Genome Biol.* **17**, 205 (2016).](https://www.zotero.org/google-docs/?4Us6eu)

[4. Himberg, J. & Hyvarinen, A. Icasso: software for investigating the reliability of ICA estimates by clustering and visualization. in *2003 IEEE XIII Workshop on Neural Networks for Signal Processing (IEEE Cat. No.03TH8718)* 259–268 (2003). doi:10.1109/NNSP.2003.1318025.](https://www.zotero.org/google-docs/?4Us6eu)

[5. Saelens, W., Cannoodt, R. & Saeys, Y. A comprehensive evaluation of module detection methods for gene expression data. *Nat. Commun.* **9**, 1090 (2018).](https://www.zotero.org/google-docs/?4Us6eu)

[6. Van Rossum, G. & Drake, F. L. *Python 3 Reference Manual*. (CreateSpace, 2009).](https://www.zotero.org/google-docs/?4Us6eu)

[7. Mölder, F. *et al.* Sustainable data analysis with Snakemake. *F1000Research* **10**, 33 (2021).](https://www.zotero.org/google-docs/?4Us6eu)

[8. McKinney, W. Data Structures for Statistical Computing in Python. *Proc. 9th Python Sci. Conf.* 56–61 (2010) doi:10.25080/Majora-92bf1922-00a.](https://www.zotero.org/google-docs/?4Us6eu)

[9. Harris, C. R. *et al.* Array programming with NumPy. *Nature* **585**, 357–362 (2020).](https://www.zotero.org/google-docs/?4Us6eu)

[10. Hunter, J. D. Matplotlib: A 2D Graphics Environment. *Comput. Sci. Eng.* **9**, 90–95 (2007).](https://www.zotero.org/google-docs/?4Us6eu)

[11. Waskom, M. L. seaborn: statistical data visualization. *J. Open Source Softw.* **6**, 3021 (2021).](https://www.zotero.org/google-docs/?4Us6eu)

[12. Pedregosa, F. *et al.* Scikit-learn: Machine Learning in Python. *J. Mach. Learn. Res.* **12**, 2825–2830 (2011).](https://www.zotero.org/google-docs/?4Us6eu)

[13. Virtanen, P. *et al.* SciPy 1.0: fundamental algorithms for scientific computing in Python. *Nat. Methods* **17**, 261–272 (2020).](https://www.zotero.org/google-docs/?4Us6eu)

[14. R: The R Project for Statistical Computing. https://www.r-project.org/.](https://www.zotero.org/google-docs/?4Us6eu)

[15. Gu, Z., Gu, L., Eils, R., Schlesner, M. & Brors, B. circlize implements and enhances circular visualization in R. *Bioinformatics* **30**, 2811–2812 (2014).](https://www.zotero.org/google-docs/?4Us6eu)

[16. Gu, Z., Eils, R. & Schlesner, M. Complex heatmaps reveal patterns and correlations in multidimensional genomic data. *Bioinformatics* **32**, 2847–2849 (2016).](https://www.zotero.org/google-docs/?4Us6eu)

[17. Strimmer, K. fdrtool: a versatile R package for estimating local and tail area-based false discovery rates. *Bioinformatics* **24**, 1461–1462 (2008).](https://www.zotero.org/google-docs/?4Us6eu)

[18. Wickham, H. *et al.* Welcome to the Tidyverse. *J. Open Source Softw.* **4**, 1686 (2019).](https://www.zotero.org/google-docs/?4Us6eu)
